# RNAi Screen for NRF2 Inducers Identifies Targets That Rescue Primary Lung Epithelial Cells from Cigarette Smoke Induced Radical Stress

**DOI:** 10.1371/journal.pone.0166352

**Published:** 2016-11-10

**Authors:** Frances-Rose Schumacher, Steffen Schubert, Michael Hannus, Birte Sönnichsen, Carina Ittrich, Stefan Kreideweiss, Thimo Kurz, Jörg F. Rippmann

**Affiliations:** 1 Boehringer Ingelheim Pharma GmbH & Co. KG, Research Germany, 8400, Biberach a. d. Riss, Germany; 2 Cenix BioScience GmbH, 01307, Dresden, Germany; 3 MRC Protein Phosphorylation and Ubiquitylation Unit, The Sir James Black Center, University of Dundee, Dow Street, Dundee, DD1 5EH, United Kingdom; Central Michigan University College of Medicine, UNITED STATES

## Abstract

Chronic Obstructive Pulmonary Disease (COPD) is a highly prevalent condition characterized by inflammation and progressive obstruction of the airways. At present, there is no treatment that suppresses the chronic inflammation of the disease, and COPD patients often succumb to the condition. Excessive oxidative stress caused by smoke inhalation is a major driving force of the disease. The transcription factor NRF2 is a critical player in the battle against oxidative stress and its function is impaired in COPD. Increasing NRF2 activity may therefore be a viable therapeutic option for COPD treatment. We show that down regulation of KEAP1, a NRF2 inhibitor, protects primary human lung epithelial cells from cigarette-smoke-extract (CSE) induced cell death in an established *in vitro* model of radical stress. To identify new potential drug targets with a similar effect, we performed a siRNA screen of the ‘druggable’ genome using a NRF2 transcriptional reporter cell line. This screen identified multiple genes that when down regulated increased NRF2 transcriptional activity and provided a survival benefit in the *in vitro* model. Our results suggest that inhibiting components of the ubiquitin-proteasome system will have the strongest effects on NRF2 transcriptional activity by increasing NRF2 levels. We also find that down regulation of the small GTPase Rab28 or the Estrogen Receptor ESRRA provide a survival benefit. Rab28 knockdown increased NRF2 protein levels, indicating that Rab28 may regulate NRF2 proteolysis. Conversely ESRRA down regulation increased NRF2 transcriptional activity without affecting NRF2 levels, suggesting a proteasome-independent mechanism.

## Introduction

COPD is a major mortality factor worldwide and characterized by a slowly progressive increase of airflow obstruction associated with emphysema and chronic bronchitis[1]. COPD carries a lifetime risk of approximately 25% for both men and women[2] and by the year 2030 it is predicted to be the fourth most common cause of death worldwide [3]. While COPD is provoked by air pollution, cigarette smoking is responsible for 90% of all cases of COPD [4]. The smoke of a cigarette contains more than 4500 separate compounds that exert massive modifications on biomolecules due to both their oxidative capacity and free radical reactivity[5,6]. One major factor contributing to the development of COPD is the misbalance of oxidative burden delivered by cigarette smoke and antioxidants in the lung, resulting in increased oxidative stress in COPD patients[7]. Increased oxidative stress occurs when epithelial lung compartments sense reactive oxygen/nitrogen species (RONS), resulting in the up-regulation of several detoxifying mechanisms [8]. The ubiquitously expressed protein NRF2 is the major transcription factor regulating the expression of phase 2 detoxifying enzymes and anti-oxidative stress proteins [9] [10]. NRF2 activates gene expression by binding to promoter elements known as antioxidant response elements (ARE) or electrophile response elements (EpRE)[11]. The levels of active NRF2 within a cell are controlled by ubiquitin-mediated proteasomal degradation. Under non-stressed and homeostatic conditions, NRF2 is constitutively degraded; this degradation is enabled through binding to KEAP1, which serves as a substrate-adaptor for an E3 ubiquitin-ligase[12]. This E3 enzyme is a Cullin-RING ligase consisting of an active core complex of Cullin3 (CUL3) and RBX1[13]; CUL3 binds to KEAP1 and KEAP1 recruits NRF2 for ubiquitination. The CUL3-KEAP1 E3 ligase complex ligates multiple ubiquitin molecules onto NRF2, targeting NRF2 to the 26S-proteasome where it is degraded [14–16]. Under conditions of oxidative or electrophilic stress, KEAP1 acts as a ‘stress sensor’ as covalent modifications of various cysteine residues in KEAP1 induce conformational structural changes, impeding NRF2 recruitment to the ubiquitylating-complex, and NRF2 protein levels are stabilized[17,18]. NRF2 then translocates to the nucleus and activates the anti-oxidative stress response of the cell through transcriptional regulation.

The expression level of the NRF2-controlled genes NQO-1, HO-1 and GPX2 are reduced in patients with severe COPD [19] and genetic depletion of NRF2 increases the susceptibility of mice to cigarette-smoke as well as elastase-induced inflammation and emphysema development[20–22]. Consistently, an over-activation of NRF2 by a lung-selective depletion of KEAP1 attenuates acute cigarette-smoke induced inflammation and reduces oxidative stress[23]. A similar protective effect was obtained by treating mice with the NRF2-inducer CDDO-imidazolide [24] and data from clinical samples as well as from animal experiments show that the NRF2/KEAP1 equilibrium is often disturbed in COPD patients [19]. This could at least in part be due to the fact that ubiquitinated proteins, such as NRF2, are sequestered in aggresomes in COPD subjects, possibly reducing the amount of available NRF2 in the cell [25] [26]. However, even though to a lesser degree, the NRF2 pathway can still be activated in this case, suggesting that not all NRF2 is aggregated and that boosting the activity of remaining NRF2 may restore activity to normal levels [25]. Together, these findings underscore the importance of the NRF2 system to the lung and provide compelling evidence that the up-regulation of NRF2 activity could be greatly beneficial for COPD patients.

Our study now further confirms that up-regulation of NRF2 provides a significant survival benefit to primary human lung epithelial cells in an *in vitro* model. In order to identify potential therapeutic targets that protect lung eptithelial cells from environmental radical stress, we performed an siRNA screen of 4995 genes considered “druggable” (e.g. genes coding for kinases, GPCRs, enzymes, receptors, channels etc. or otherwise examples of proteins that may allow pharmacological modulation of their function). We could identify genes that when depleted, result in an up-regulation of NRF2 activity, a concomitant increase of expression of the NRF2 target NQO1 and promote survival of primary lung epithelial cells when exposed to CSE. These genes provide a starting point for future research into potential new treatments for COPD.

## Materials and Methods

### Cloning of NRF2 reporter construct

The plasmid pTKLN-hNQ was a kind gift from Bastian Hengerer, BI Pharma GmbH Co KG. The construct was responsive to the induction of NAD(P)H:quinone oxidoreductase type I (hNQO-1) expression due to the insertion of a NRF2 promoter response element upstream of the minimal thymidine kinase promoter which drives the expression of the firefly luciferase. The negative control vector pTK-luc2 was generated by a 35 bp deletion of the NRF2 response element with a NotI SalI (Gibco BRL) double restriction and relegation after Klenow fragment treatment.

### Cell culture and transfection protocols for HEK293 and HBEpC cultures

The cell line HEK293 (ATCC, CRL-1573™) and derivates thereof were cultured in DMEM (Invitrogen) supplemented with 10% FCS (GibcoBRL). Human bronchial epithelial primary cells (HBEpC, Promocell) were maintained in special HBEpC medium (Promocell). All cell lines and isolates were exponentially grown at 37°C in a 5% CO_2_ atmosphere.

The NRF2 reporter cell line HEK293-hNQ was generated from HEK293 cell cultures by transfection of plasmid pTK-luc2 (HEK293-hNQ-del) or pTKLN-hNQ (HEK293-hNQ) with Lipofectamine2000 (Invitrogen) according to manufacturer´s protocols. After 24 h the chromosomal integration of the reporter constructs was selected by the addition of G418 at 1 mg/mL concentration, which was included in all further culture steps. Single clones were isolated from the pTKLN-hNQ transfected (HEK293-hNQ) and pTK-luc2 transfected (HEK293-hNQ-del) HEK293 cell pools by limited dilution.

For compound treatment HEK293-hNQ cells were seeded in 100 μL medium on poly-D-lysin coated 96 well plates (Biocat #356690) at 3×10^4^ cells per wells 24 h prior to the experiment to become adherent and 70% confluent. Compounds were prepared as DMSO stock solutions and diluted in fresh, prewarmed medium for the treatment of cell cultures. A final DMSO concentration of 0.1% was adjusted for all compound dilutions. Prior to treatment, cells were washed with prewarmed medium and incubated with the compound dilutions for 24h.

For all experiments siRNAs were purchased from Ambion (order numbers see S1 Table) and prepared according to manufacturer’s instructions. As negative control the OnTarget plus siCONTROL non-targeting siRNA (D-001810-01-20, Dharmacon) was used in all 96-well format experiments. As positive control for the activation of NRF2 transcription factor activity three siRNAs against KEAP1 (#s18983, #s18981, #19864, Ambion) were pooled and analyzed within one well.

The HEK293 cell cultures and derivates thereof were reverse transfected on poly-D-lysin coated 96-well plates (BioCoat): For the preparation of the liposomal siRNA complex, 0.225 μL/well of Dharmafect 1 reagent was diluted with DCCR buffer to give 20 μL and incubated for 30 min in the cell culture plates with 6.25 pmol/5μL siRNA mixture. The cell suspension of 3×10^4^ cells/100 μL in growth medium supplemented with 10% FCS was added to each well and incubated for 48 h under standard culturing conditions.

The HBEpC cell cultures were reverse transfected with the use of RNAiMax (Invitrogen): For the preparation of the siRNA mixture, 0.313 μL of siRNA stock solution (20 μM) was added to medium without serum supplement to give 5 μL of siRNA mixture. The liposomal reagent was prepared by the addition of 0.4 μL of RNAiMax to medium without serum supplement to give a final volume of 25 μL. The siRNA mixture and the liposomal reagent were mixed and incubated at room temperature for 20 min in the 96-well plate prior to the addition to an HBEpC cell suspension of 2×10^4^ cells/100 μL. Cultures were further incubated under standard culturing conditions for 48 h until stimulation with CSE as described below.

For the high-throughput screening 3×10^3^ cells/30 μL were seeded on 384 well 4titude collagen-1 coated optical plates. After 24 h cells were transfected with 30 nM of pools (three siRNAs for each gene, 4995 genes in total for first screening round) using 0.04 μL Dharmafect 2 (Thermo Fischer). Cultures were further incubated under standard culturing conditions for 48 h.

### RNA isolation and quantitative RT-PCR expression analysis

For the gene expression analysis total RNA was isolated from cell culture lysates according to the RNeasy protocol (Qiagen). Briefly, cells were rinsed with PBS and lysed directly in the 96-well plate with 150 μL of RLT-lysis buffer. Total RNA was precipitated by the addition of 1 Vol. 70% ethanol. The precipitate was loaded and centrifuged on RNeasy spin column (10 000×g, 2 min, Qiagen), DNAse treated and eluted in 50 μL DEPC-H_2_O. Total RNA concentration was quantified by SYBR green II (Sigma) assay (D.Schmidt & J.D.Ernst (1995), Anal. Biochem. 232, 144–146). The purified total RNA was stored at -20°C. The gene expression levels were determined by TaqMan® analysis in a 7900HT Sequence Detection System (Applied Biosystems) using High capacity cDNA Archive Kit (Applied Biosystems) for the reverse transcription and PCR amplification in ABI PRISM 384 well optical reaction plate (Applied Biosystems). Gene specific probes were labeled with 6-FAM™ and TAMRA for internal quenching. Sequences of forward-, reverse- and probe-oligonucleotides were for hNQO1 5´-CAGATATTGTGGCTGAACAAAAG-3´, 5´-CATCAGCATTCTCTCCCATGGT-3´, 5´-AAAACCCAGAGAACAGCTACCACCTTTACAGC-3´ and for the house-keeping gene RNA-Polymerase-2 5´-GCCAAAGACTCCTTCACTCACTGT-3´, 5´-TTCCAAGCGGCAAAGAATGT-3´, 5´-TGGCTCTTTCAGCATCTCGTGCAGATT-3´. For these primer and probe combinations, the Mn^+2^-concentrations were optimized to reach nearly 100% amplification efficiency. Relative quantities of expression levels were determined by comparison of ct-values with a dilution series of a standard total RNA samples and normalized for the RNA polymerase-2 quantity.

### Luciferase activity determination

The enzymatic activity of luciferase in the lysates of HEK293 cell cultures was used as readout for NRF2 transcription factors activity. Under standard and RNAi screening conditions, cells were lysed 48 h post transfection by the addition of 20 μL of One-Glo luciferase reagent (Promega). An aliquot of 25 μL was transferred to white 384-well plates and luminescence generated by luciferase activity was determined 30 min later on a Wallac Victor2 (Perkin Elmer) luminescence reader.

### Nuclear morphometry assay

Nuclear DNA staining was used for the detection of proliferation and as indicator of apoptosis. Two days after transfection of siRNAs, cell cultures were fixed and stained with 4% PFA, 1 g/mL Hoechst33342 (final concentrations) at RT. Images (4 sites per well) were acquired on an ImageXPress Micro automated microscope (MDC) with a 10× objective. Quantitative automated image analysis was performed using automated eCognition (Definiens) software. CENIX algorithms for segmentation and classification of nuclei were customized for HEK-hNQ cells. The number of nuclei per image field was used as a measurement for culture proliferation and the fraction of nuclei with condensed chromatin per image field was used as an indicator of apoptosis.

### High throughput RNAi screening workflow and calculation of results

For the high-throughput RNAi screening test siRNAs pools were randomly distributed on 19 source plates. As positive controls pools of 3 siRNAs for KEAP-1 (#s18981, #s18983, #19864, Ambion), CUL-3 (#s16048, #s16049, #s16050, Ambion) and PLK-1 (#s448, #s449, #s450, Ambion) were selected. Negative controls were pools of three phenotypically neutral siRNAs (pool1: #10278241, #102782456, #102782457, pool2: #10278240, #10278249, #10278259, pool3: #10278250, #10278257, #10278265; Cenix) and transfection reagent-only samples. Controls were arranged as intra-plate quadruplicates on each source plate. siRNA pools from each source plate were transferred to yield six replicate experimental plates per source plate. 48 hours later, the first plate triplicate was fixed and stained for automated microscopy analysis, while cells in the second plate triplicate were lysed and used in luciferase assay. Experimental plates from 2–7 source plates were processed simultaneously to maximize throughput.

For microscopic analysis, image QC automatically excluded all image data with suboptimal focus, high background or gross artifacts. Number of nuclei and nuclear condensation indices from the four image fields were averaged for each well. For each experimental plate, well averages of microscopy or single-well luciferase results were normalized to the average of the 12 intra-plate negative control wells (intra-plate normalization). Means and standard deviations of the normalized well averages were calculated between inter-plate replicates. For data visualization and hit selection Spotfire DXP was used.

### CSE stress induction and cellular vitality determination

Cigarette smoke extract (CSE) was used as irritant for cell cultures of HEK293-hNQ and primary bronchial epithelial cells (HBEpC). The extract was always freshly prepared according to standard procedures. Briefly, the smoke of 5 Roth-Händle cigarettes per 50 mL medium was sucked with constant vacuum through the culture medium (lacking phenol red). The CSE was sterile filtered (0.45 μm) and regarded as 100% CSE medium. Dilutions were performed with normal growth medium (lacking phenol red) as described in results section and cell cultures were incubated for 16 h. Prior to harvest, cells were washed with PBS and further processed either for RNA extraction or the determination of intra-cellular ATP levels with the CellTiter-Glo Luminescent Cell Viability Assay (Promega) as marker for vitality.

For the comparison of CSE tolerance induction, HBEpC cultures were reverse transfected, incubated for 48 h and stimulated with 3% CSE medium for additional 16 h. Each siRNA was analyzed as intra-plate duplicates. Experiments were considered to be valid if within one plate means of KEAP1 knock down samples showed at least 3-fold higher ATP-levels as means of siControl treated cultures. Experiments were repeated until at least 4 biological replicates passed this quality control parameter. For the statistical analysis mean ATP-levels of test siRNAs were normalized to mean ATP-levels of siControl treated samples of the same plate. Means of biological replicates were calculated from these normalized values and significant activation of CSE tolerance was assessed by two-sided students T-test of sample means against normalized means of transfection reagent treated controls.

### NRF2 stability assays

All siRNA oligonucleotides were purchased from Thermo Scientific Dharmacon, we used ON-TARGETplus siRNA SMARTpool siRNA again the human proteins for targeted knock-down (Catalogue numbers: KEAP1 LU-012453-00, CUL3 LU-010224-00, ESRRA LU-003403-0002, PLCH2 LU-027529-01, PSMC2 LU-008180-01, PSMC4 LU-009261-01, PSMD6 LU-021249-02, SUV39H1 LU-009604-00, RAB28 LU-008582-00). The scrambled negative siRNA control was purchased Thermo Scientific Dharmachon also (ON-TARGET plus Non-targeting Pool D-001810-10) Transfections were performed using 10 nM oligonucleotide and Lipofectamine (Life Technologies) transfection reagent according to the manufacturers protocol in a 6-well plate format. Cells were lysed in 350 μL lysis buffer (50 mM Tris/HCl (pH 7.5), 0.15 M NaCl, 1 mM EGTA, 1 mM EDTA, 1 mM Na3VO4, 50 mM NaF, 5 mM Na4P2O7, 0.27 M sucrose, 1% (w/v) Nonidet P40, 1 mM benzamidine, 0.1 mM PMSF, 0.1% 2-mercaptoethanol) after 96 h transfection. The lysates were clarified by centrifugation at 4°C and quantified using Bradford assay. For each condition 50 μg clarified lysate was boiled in sample buffer and subjected to SDS PAGE and transferred onto nitrocellulose membranes. Membranes were blocked for 1 h in TBST containing 10% (w/v) dried skimmed milk and then incubated overnight at 4°C in primary antibody (NRF2: Abcam ID: ab62352, diluted 1:1000, Actin: SigmaAdlrich diluted 1:5000). Membranes were then washed five times in TBST and incubated for 1 h at room temperature with the appropriate secondary HRP-conjugated antibody. Membranes were washed a further five times in TBST, and the signal detected following HRP activation with enhanced chemiluminescence reagent (GE Healthcare). Signal was detected using a GelDoc.TM Molecular Imager (Biorad XR+ Imaging System) and images were processed and quantified using Image Lab 3.0. NRF2 band intensities were normalized to actin band intensities. To enable the relative contribution of knocking down each gene by siRNA had on NRF2 levels, these were then expressed relative to the control siRNA NRF2 level. This experiment was performed in triplicate enabling differences to be statistically powerful. Data was analyzed using Prism6 (GraphPad Software, Inc.) and paired-T-tests were performed to determine significance. (p<0.05).

## Results

### Upregulation of NRF2 activity provides a survival benefit in an in vitro model of radical stress

**Fig 1:**
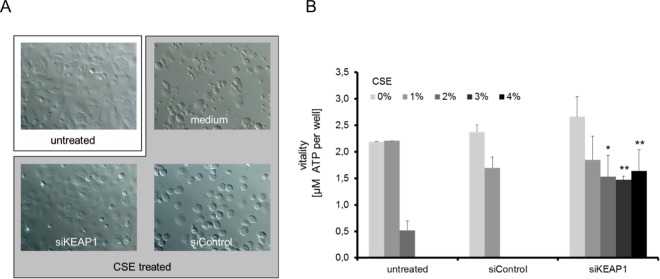
Induction of CSE stress tolerance in HBEpC cell cultures by KEAP1 knock down. Cell cultures of primary human bronchial epithelial cells were transfected for 48h with positive and negative control siRNAs prior to stimulation with 3% CSE-conditioned medium. After stimulation for 24h cells were analyzed either by Zeiss Axioplan (20× magnification) microscopy (A) or for their vitality by the Cell Titer Glo assay (B). Downregulation of KEAP1 greatly improves the survival of cells after CSE exposure. An ATP standard was used for the calculation of ATP concentration per well. Data are represented as means of 3 independent biological replicates +SD. Significant differences of siKEAP1 compared to siControl treated samples are highlighted (* p≤0.05, ** p≤0.01, students T-test).

COPD most commonly affects people with a history of cigarette smoking and the acute effects of cigarette smoke can be studied *in vitro* by exposing cells to the chemicals contained in cigarette smoke. This is most commonly done by means of cigarette-smoke-extract (CSE), which is generated by passing cigarette smoke through cell growth medium to dissolve its soluble compounds. Subsequently, cells are exposed to the medium and their response is measured. To determine the effect of NRF2 up-regulation on the survival of lung cells after CSE exposure, we used primary human bronchial epithelial cells (HBEpC, PROMOCELL) and first exposed them to CSE to determine how the cells tolerate this stress. As a consequence of the treatment cells became notably more round in shape and died after exposure to 3% CSE (Fig 1A). It was previously shown that the lung-specific deletion of KEAP1 in mice leads to increased NRF2 activity, which reduces oxidative stress and attenuates inflammation after cigarette smoke exposure[23]. We next tested if this holds true in our assay. Indeed, down-regulation of KEAP1 similarly provided a survival benefit to the HBEpCs in culture, as cells treated with KEAP1 siRNA prior to CSE exposure maintained their epithelial morphology and survived the treatment (Fig 1A). When quantified using the intra-cellular ATP levels as a measure of viability, we found that primary human lung epithelial cells were able to withstand up to 2% CSE without any significant drop in viability, however when exposed to CSE levels above 2% cells died quickly (Fig 1B). Dramatically, when KEAP1 was down regulated, cells survived at CSE concentrations of >4% (Fig 1B). This major increase in viability adds to a large body of evidence suggesting that the up regulation of the NRF2 pathway is beneficial to cells after cigarette smoke exposure, which further motivated us to explore the possibility of identifying drug targets that result in NRF2 activation in the lung.

### Establishment of a NRF2 reporter cell line suitable for high throughput siRNA screening

We decided to perform a siRNA screen using a HEK293 cell line that reports on NRF2 activity by expressing the firefly luciferase gene under the NRF2-responsive NQO1 promoter (HEK293-hNQ), which was integrated into the genome of the cells. As a negative control cell line we used cells carrying the same construct, which was made non-sensitive to NRF2 induction due to the deletion of a 35 bp region of the NRF2 response element (HEK293-hNQ-del).

**Fig 2:**
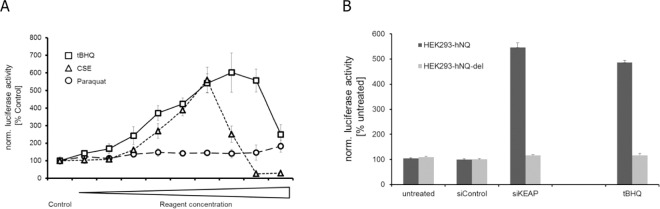
Activation of the NRF2 transcription factor reporter system by different agents of radical stress or KEAP1 modifier. (A) Cell cultures of HEK293-hNQ cells were treated for 24 h with tBHQ (3, 6, 12, 18, 25, 37, 50, 75, 100 [μM]), CSE (0.04, 0.08, 0.16, 0.32, 0.63, 1.25, 2.5, 5, 10 [% (v/v)]) or Paraquat (25, 50, 75, 100, 125, 150, 200, 250, 300 [μM]). Luciferase activity was expressed as % of untreated samples (Control). (B) Cultures of HEK293-hNQ and HEK293-hNQ-del cells were transfected with positive and negative control siRNAs and lysed 48 h later for the determination of luciferase activity. Treatment of these cells with the NRF2 activator tBHQ at 50μM was performed for 24h. All results were normalized to untreated HEK293-hNQ samples. Values are represented as means of independent biological experiments ± SD (n = 6).

We confirmed the sensitivity of the system using known NRF2 inducers; measuring the induction of the luciferase gene after stimulation with cigarette-smoke extract (CSE) or the compound Paraquat, which generates oxygen radicals (Fig 2A). Paraquat gave only a minor induction of luciferase activity (1.8 fold at highest concentration of 300 μM Paraquat) and showed a dose dependent toxicity as seen by cell rounding and detachment from the culture plates (data not shown). CSE, however, induced luciferase activity up to 5.6-fold at 1.25% CSE concentrations, while higher CSE concentrations were also toxic for the cells (Fig 2A). To further define our reporter system, we exposed the cells to the compound tBHQ, which is known to modify KEAP1, resulting in a release of NRF2 from the inhibitory complex and the activation of NRF2 dependent gene transcription[27]. Consistent with this effect, the HEK293-hNQ cells responded in a dose-dependent manner to the treatment of tBHQ, with a maximum of 6-fold induction of luciferase activity at a 50 μM concentration of tBHQ (Fig 2A). Comparable activation of the luciferase reporter was seen after siRNA downregulation of KEAP1 (Fig 2B). The control cultures of HEK293-hNQ-del cells did not respond to the compound treatment or the siRNA knock down of KEAP1 (Fig 2B), demonstrating that the HEK293-hNQ cell line accurately reports on NRF2 transcriptional activity while also confirming this cell line’s utility for the siRNA screen.

### Primary RNAi screening of 4995 genes identifies 219 potential activators of NRF2

**Fig 3:**
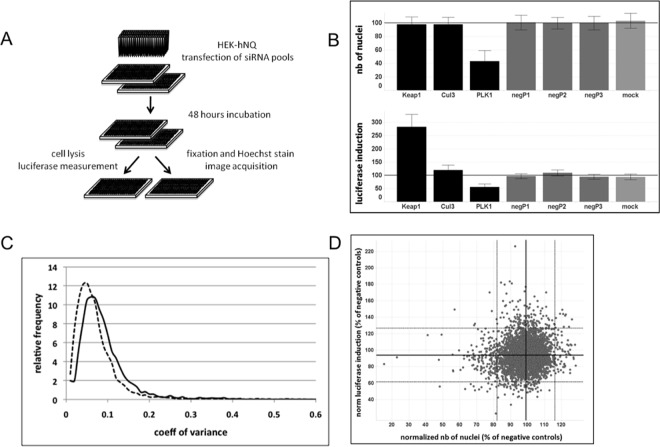
RNAi screen to detect activators of NRF-2. (A) HEK-hNQ cells were transfected with siRNA pools (3 siRNAs per gene) in 384-well format. 48 hours post transfection, cells on three replicate plates were lysed and luminescence was measured. Cells on identical triplicate plates were stained with Hoechst dye and images were acquired using an automated microscope. (B) Performance of control siRNA pools. Average and standard deviations of normalized values for control siRNA pools on all plates. (C) Frequency distribution of coefficients of variance between three replicates indicates high reproducibility of screen results. Solid line, normalized luminescence measurements, dashed line, normalized number of nuclei. (D) Performance of control siRNA pools. Average and standard deviations of normalized values for control siRNA pools on all plates.

We conducted a RNAi screen of 4995 druggable genes in the HEK293-hNQ reporter cell line to identify genes that are involved in the regulation of NRF2 activity (see Fig 3A for a workflow of the screen and S1 Table). The strongest response obtained with known inducers of NRF2 during testing of the cell line was a 5-fold activation of the system, and we set this as our maximal response (Fig 2A and 2B). Briefly, we transfected cells in a 384-well format with siRNA pools consisting of three gene-specific siRNAs and measured luciferase induction 48 hours after transfection. To assess possible effects of the siRNA transfection on cell proliferation, satellite plates transfected with the same siRNA pools were stained with Hoechst dye and cell numbers in each well were recorded using an automated microscope (Fig 3A).

We normalized the data to the average of three negative control siRNA pools positioned on each screening plate that were selected for neutral behavior towards NRF2 activity and cell proliferation. As expected, no significant effect on luciferase induction or cell number was observed with the negative control pools (Fig 3B).

A siRNA pool directed against KEAP1 served as positive control. This pool increased luciferase activity by approximately 3-fold under screening conditions with high reproducibility and without impacting cell proliferation (Fig 3B). A siRNA pool directed against CUL3, another component of the E3 complex that targets NRF2 for proteasomal degradation, showed mild effects on luciferase activity. The average luciferase induction by CUL3 knockdown throughout the screen was 116 ± 6% compared to negative controls (Fig 3B). This pool was used as a weak positive control.

As a control for the validity of the cell proliferation read-out, a siRNA pool directed against PLK1, a gene that plays a major role in mitosis, was included. Knockdown of PLK1 reproducibly reduced the number of nuclei by approximately two-fold (Fig 3B). Importantly we observed that all control siRNA pools in the screen performed according to expectations and increased confidence in the validity and reproducibility of the assay results.

The siRNA screen was performed in triplicate with a high degree of consistency between the three datasets. For 80% of the data, coefficients of variance were below 10% for both luciferase induction and proliferative index (Fig 3C). Most data points were distributed evenly in a cluster around the values for the negative control pools, indicating that effects on cell proliferation and activation of luciferase are rare and specific events (Fig 3C and 3D). Relatively few siRNA pools increased luciferase activity by a wide margin and the positive control siRNA pool against KEAP1 remained the strongest hit of all genes tested (S1 Table). This suggests that the assay is highly specific and further provides confidence in hits showing a strong phenotype.

Knockdown of some genes led to intermediate increases in luminescence and these may also be genuinely involved in NRF2 activation. We therefore decided to apply simple and inclusive criteria in the choice of genes for further validation. Hits were defined as genes whose knockdown resulted in an increase in luciferase activity greater than two standard deviations from the mean of all siRNA pools tested. This threshold corresponded to approximately 126% luciferase activity in comparison to negative control wells. 159 genes passed this hit selection threshold.

To account for effects on cell proliferation that may mask luciferase induction, we also normalized luciferase data to the number of cells in each well. By this metric, 154 genes fell above two standard deviations from the mean, 94 of which overlapped with the genes identified in the hit list without normalization for proliferation. The list of genes that were taken forward for confirmation in pass 2 therefore consisted of 219 genes (S2 Table)

### Deconvolution of siRNA pools

In the screen each gene was targeted by a pool of three different siRNAs. This approach is thought to afford high efficacy of knockdown, as several siRNAs against the same mRNA will increase the likelihood of specific gene knockdown, while off-target effects by each individual siRNA may be diluted [28]. However, the observed phenotype may still be caused by an off-target effect of any one of the three siRNAs in the pool. We thus tested the contribution of each siRNA to the observed phenotype individually. In addition to providing a confirmation of pass 1 results, this strategy allowed the stratification of hits into those that are mainly due to the action of a single siRNA and those whose phenotype is corroborated by several siRNA against the same gene.

**Fig 4:**
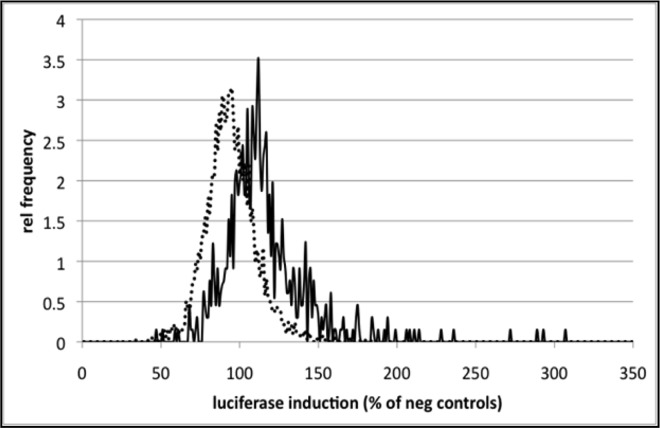
Confirmational screen with 219 genes using 3 individual siRNAs against each gene. Enrichment for luciferase-inducing genes. Frequency distribution of luciferase induction for all siRNA pools screened in pass 1 (dashed line) and the 657 individual siRNAs used in pass 2 (solid line).

The overall set-up of the pass 2 screen using three individual siRNAs against each of the 219 hit genes was generally the same as in pass 1. As expected, the distribution of data points was moderately shifted toward luciferase induction in this round of experiments (Fig 4). Consistent with results in pass 1, reproducibility between replicates was high. Comparing the performance of siRNA pools and individual siRNAs, for the majority of the tested genes, differences in luciferase induction were minor between pools and the best individual siRNA against the same gene. In 31/219 cases, pools performed significantly weaker than top individual siRNAs at the 5% confidence level, in 22/219 of cases, the effect of pools was significantly stronger.

In keeping with hit selection criteria in pass 1, we defined as hits all siRNAs that resulted in luciferase activity equal to or greater than 130% compared to negative controls. By this metric, approximately half of pass 1 hits were confirmed (107/219). 88 of these genes were validated by one of the three siRNAs. The effect of 18 additional genes was corroborated by two siRNAs against the same gene, while one hit was confirmed by all three siRNAs. Importantly, two individual siRNAs each against positive controls KEAP1 and CUL3 strongly induced luciferase activity.

An additional 32 genes passed the confirmation threshold when luciferase data was normalized to cell number within the well resulting in 139 confirmed hits in total (S3 Table).

The three single siRNAs of the 139 hits from the pass 2 screening campaign were analyzed a second time in 96-well plates under reverse transfection conditions in HEK293-hNQ cell cultures. The luciferase results derived from the HEK293-hNQ cell cultures in 96-well plates correlated with the previous pass 2 experiment (r = 0.673, p<0.0001, data not shown), confirming the robustness of the assay principle. Out of the 417 siRNAs tested (S4 Table), 150 activated luciferase in the HEK293-hNQ cell cultures equal or greater than 130% compared to negative controls. Out of these, 77 genes were represented by single siRNA hits, 23 genes were represented by two independent siRNAs and nine genes were represented by three independent siRNAs. Thus, of the initial 139 hits, only 32 were confirmed with at least two independent siRNAs.

### Analysis of endogenous hNQO1 gene expression activation by hit genes of the primary RNAi screen

**Fig 5:**
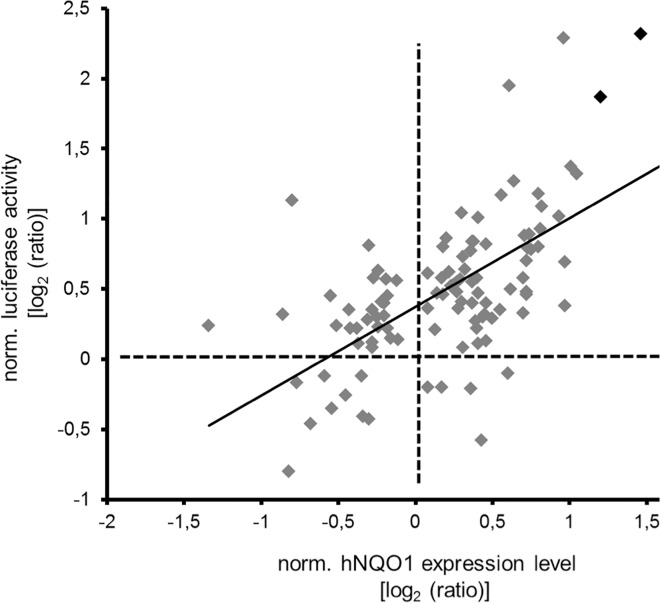
Activation of the NRF2 transcription factor reporter system correlates with the induction of the NRF2 responsive gene NQO1. siRNAs of hit candidates of the primary RNAi screen were transfected into HEK-hNQ cells and luciferase activity or hNQO1 mRNA levels were determined 48 h post transfection. The log2 ratios of specific luciferase activity were plotted versus log2 ratios of normalized expression levels of human NQO1 of identical siRNA treated samples. Only results of significant differences (p≤0.05) in both readouts are shown. SiKEAP1 treated samples are highlighted in black.

To further validate the 139 hits, we tested whether their down-regulation would result in the up regulation of endogenous hNQO1 mRNA by qRT-PCR. In our initial screen the hNQO1 promoter drove the luciferase reporter and we now wanted to test whether the transcription of endogenous hNQO1 was also up regulated by the identified hits. This approach allowed us to exclude genes that stabilize luciferase independently of NRF2 transcriptional activity. As a positive control we again utilized KEAP1 siRNA, which induced the hNQO1 mean gene expression 2.75-fold compared to control siRNA or untreated HEK293-hNQ cultures. The hNQO1 expression analysis correlated well with the luciferase induction analysis (r = 0.548, p<0.0001) of the HEK293-hNQ cell cultures (Fig 5). From the 414 tested siRNAs (not including the KEAP1 and control siRNAs), 41 siRNA increased the hNQO1 mRNA levels in HEK293-hNQ cell cultures significantly (p<0.05) equal or greater than 130% compared to negative controls. These siRNAs represented 35 genes, 29 genes were represented by single siRNA hits, and six genes were represented by two siRNAs. KEAP1 was the only gene where three independent siRNAs upregulated hNQO1 significantly (S4 Table).

### Analysis of CSE induced stress tolerance by downregulation of selected NRF2 activators in primary bronchial epithelial cell cultures

The initial rationale of our screen was based on the hypothesis that stabilization of NRF2 may provide a survival benefit in COPD patients. To confirm this assumption, the CSE survival assay was developed as a screening protocol for the identification and confirmation of siRNAs as survival factors. Unfortunately, the assay was not robust enough for screening purposes and several plates did not pass quality control due to poor performance of positive and negative controls. As a result, only 48 genes could be analyzed with regard to their siRNA knock down providing a CSE tolerance to primary HBEpCs (S5 Table). Of the tested siRNAs, that passed quality control, 12 siRNAs improved survival of HBEpCs significantly and three genes were represented by two independent siRNAs (CUL3, PSMC2, PSMD6). Of these confirmed genes, the three proteasomal subunits PSMC4, PSMC2 and PSMD6, as well as the E3 ubiquitin ligase CUL3, are involved in directly regulating NRF2 levels by proteasomal degradation.

### Analysis of NRF2 protein levels

**Fig 6:**
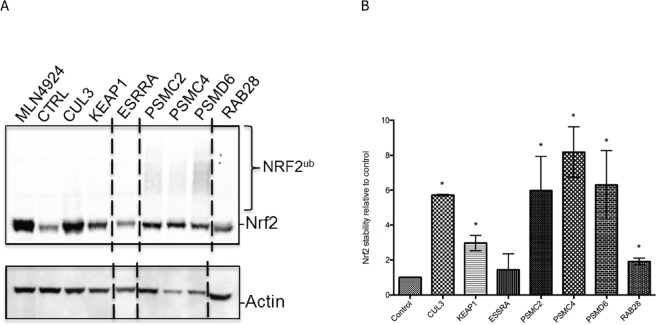
Western Blot analysis of NRF2 levels in selected screening hits. (A) Anti-NRF2 Western Blot of tissue cell lysates after siRNA to the indicated screening hits. Treatment of cells with MLN4924, a small molecule inhibitor of CUL3 and other Cullin-RING ligases, leads to a strong increase in NRF2 levels as compared to control siRNA cells (CTRL). siRNA downregulation of CUL3, KEAP1, PSMC2, PSMC4, PSMD6 and RAB28 also lead to increases in NRF2 levels. Poly-ubiquitylated species of NRF2 after downregulation of proteasomal subunits are also apparent (NRF2^ub^). A Western Blot against Actin is included as loading control. All samples shown were run on the same gel. Dashed lines indicate where lanes have been removed for clarity. (B) Quantification of NRF2 Western Blots for the indicated siRNA samples relative to siRNA control samples. Asterisk indicates significant stabilization of NRF2 (p<0.05).

Throughout our screen we assumed NQO1 expression would be regulated by increases in NRF2 protein levels. However, this effect could also be mediated without altering overall NRF2 levels. To address this, we asked whether the hits that induce CSE tolerance do so by increasing NRF2 protein levels. The four strongest hits we identified are either components of the E3 ubiquitin ligase complex that targets NRF2 to the proteasome (CUL3, KEAP1), or subunits of the proteasome itself (PSMC2, PSMC4, PSMD6), and we expected these to induce a stabilization of NRF2 when inactivated. Indeed, Western Blot analysis revealed that siRNA to these genes significantly stabilized NRF2 protein levels (Fig 6A and 6B) in a manner comparable to cells treated with MLN4924, a drug that inhibits Cullin-RING ligases through preventing their activation by the ubiquitin-like protein NEDD8 [29]. Down regulation of the proteasomal subunits also resulted in the appearance of ubiquitylated NRF2 species (Fig 6A), consistent with the constitutive ubiquitylation of NRF2 by CUL3-KEAP1 in the context of a defunct proteasome unable to perform proteolysis. As such this observation confirms the role of the proteasome in the regulation of NRF2 levels.

Two other genes we identified (ESRRA and RAB28) have no reported known link to NRF2 function, yet their down regulation activates NQO1 expression and provides a survival benefit to CSE-exposed cells, suggesting that they are genuine regulators of NRF2 transcriptional activity. Down regulation of RAB28 also increased NRF2 levels, albeit to a lesser extent than CUL3/KEAP1 or the proteasome (Fig 6A and 6B). Down regulation of ESRRA, on the other hand, had no effect on NRF2 protein levels, indicating that it regulates NQO1 transcription independently of the overall NRF2 protein abundance (Fig 6A and 6B).

## Discussion

Here we describe an unbiased siRNA screening approach to identify regulators of NRF2 transcriptional activity. Our ultimate goal was to find new potential drug targets for the treatment of COPD, and our initial screen with the druggable 4995 genes provided a list with numerous candidates. Through successive rounds of confirmatory screening, the number of genes affecting NRF2 transcriptional activity was reduced extensively. Of these genes, five are involved in the proteasomal degradation of NRF2 and their downregulation thus leads to NRF2 stabilization and induction of NRF2 transcription. This is consistent with previous findings that show that a proteostasis imbalance is involved in the pathogenesis of COPD by affecting NRF2 levels [30] and that the expression of proteasomal subunits correlates inversely with the levels of NRF2 and its transcriptional activation [31]. Importantly, down regulation ofthe identified genes provided a survival benefit to cells exposed to cigarette smoke, demonstrating their suitability as targets for COPD treatment. Of these five genes, three were subunits of the proteasome itself and inhibition of the 26S-Proteasome has already been explored therapeutically. Bortezomib and Carfilzomib, two proteasome inhibitors, are very successfully used for the treatment of multiple myeloma[32]. It is, however, doubtful that this approach would also work for COPD, as the 26S Proteasome is responsible for most cellular protein degradation and has many essential functions. Furthermore as COPD is a chronic disease, it will likely require life-long treatment and any medication would have to be well tolerated. Given the many important functions of the 26S-Proteasome, a chronic and systemic treatment with proteasome inhibitor would likely not be feasible due to the potential severity of side effects, although it may be worthwhile to explore a more localized proteasome inhibition that is restricted to the lung epithelium. A more specific inhibitor of NRF2 degradation would be desirable to circumvent the general inhibition of protein degradation and the associated side effects. Two of the other genes we identified are candidate targets for more specific degradation inhibitors. Both, CUL3 and KEAP1, are components of the ubiquitin E3 ligase that specifically attaches ubiquitin to NRF2. Ubiquitylated NRF2 is then recognized by the proteasome and degraded. Inhibition of the E3 ligase would therefore be a more specific target than general inhibition of the proteasome. However, CUL3 itself likely forms more than one hundred E3 ligase complexes through association with many different substrate adaptors, and as such inhibiting CUL3 would also affect numerous substrates in addition to NRF2. KEAP1, on the other hand, is a substrate adaptor for CUL3, and it may only target NRF2 and few additional proteins for degradation[33]. Inhibiting KEAP1 could therefore be a novel target to explore in the context of COPD treatment. However, as KEAP1 itself has no enzymatic activity, but functions by directly binding to NRF2 and recruiting it to CUL3, an inhibitor would most likely have to target the KEAP1/NRF2 interface to prevent the interaction of the two proteins. Whether this approach is feasible remains to be determined.

Aside from proteins involved in proteasomal degradation of NRF2, we also identified RAB28 and ERRα (ESRRA), two proteins currently unknown to regulate NRF2. RAB28 is a small GTPase of the RAB family, of which there are approximately 60 encoded in the human genome. RAB proteins are known to regulate membrane dependent processes, such as intracellular trafficking, but a defined function for Rab28 has not been determined[34]. Mutations in Rab28 have been found to cause two types of cone-rod dystrophy (18 and 6)[35]; the molecular mechanism underlying the disease, however, is unclear. We found downregulation of RAB28 enabled cells to withstand higher doses of CSE and led to an approximately two-fold increase in NRF2 protein levels. This is significantly less than the increase mediated by CUL3, KEAP1 and proteasome downregulation, yet the survival benefit is comparable. How RAB28 downregulation leads to NRF2 stabilisation is unclear, and it remains to be determined if it is the moderate upregulation in NRF2 levels that is solely responsible for the observed survival benefit. However, as RAB28 downregulation also leads to a significant induction of NRF2 controlled genes, it will certainly be a major contributor to the observed effect. If, like other RABs, RAB28 regulates some aspects of intracellular trafficking, it will be important to understand in the future how this process is involved in maintaining NRF2 levels. As small GTPases are good drug targets, it should be feasible to develop a RAB28 inhibitor, however, given the association of RAB28 with cone rod dystrophy, future work is required to determine if chronic treatment with such an inhibitor is a feasible therapeutic option.

We also found that downregulation of ESRRA, which encodes for the protein ERRα (estrogen-receptor related alpha) gives a significant survival benefit to cells exposed to CSE. ESRRA downregulation also induces NRF2 responsive genes, but interestingly does not lead to NRF2 stabilisation. This indicates that ERRα regulates the NRF2 transcriptional response independently of NRF2 levels. Nuclear receptors, such as ERRα, are transcription factors that are typically activated by ligand binding, but they also appear to have constitutive activities [36]. The ligand for ERRα has not yet been identified, but as many genes will likely be regulated by ERRα it is not difficult to imagine that this could include inhibitors of the NRF2 response.

Most importantly, ERRα might directly inhibit the transcription of NRF2 responsive genes, such as NQO1. Downregulation of ERRα could thus lead to the transcription of these genes even in the absence of NRF2, resulting in the observed survival benefit. A connection between ERRα and oxidative stress has been already established as previous work has shown that ERRα transcribed genes are involved in mitochondrial oxidation pathways [37]. Pharmacologically, nuclear receptors can be targeted by small molecules and ERRα inhibitors for use in experimental settings exist (Compound A; XTC790) [38,39]. It therefore appears feasible to develop small molecules targeting the protein.

Prior to any clinical development it will be important to assess our identified hits in animal models of COPD to confirm their feasibility in an organismal setting. Our results indicate the proteasome and the CUL3-KEAP1 E3 ligase complex targeting NRF2 are the most promising targets for COPD treatment. Targeting KEAP1 in particular may give the most benefit with the least side effects and could have great therapeutic potential.

## Supporting Information

S1 Table First RNAi screen in HEK-hNQ cell line on luciferase induction and nuclear condensation. Screening hits are marked in grey.
(XLSX)

S2 Table List of 219 genes that were considered as hits from the first screen and deconvoluted in a second screen with three independent siRNA molecules. (XLSX)

S3 Table Second RNAi screen in HEK-hNQ cell line on luciferase induction and nuclear condensation with three independent siRNA molecules per gene. Screening hits are marked in grey.
(XLSX)

S4 Table Third RNAi screen in HEK-hNQ cell line on luciferase induction and NQO1 mRNA regulation with three independent siRNA molecules per gene in an 96-well format. Screening hits are marked in grey.
(XLSX)

S5 Table Fourth RNAi screen in HBEpCs on the survival of CSE induced stress with three independent siRNA molecules per gene. Screening hits are marked in grey.
(XLSX)
